# Sporadic Malignant Cholera

**Published:** 1875-03

**Authors:** 


### Our Exchanges.

Sporadic Malignant Cholera.—Dr. Willis Alston, of Littleton, N. C., reports, *New York Medical Journal*, February, 1875, a case resembling malignant cholera generated *de novo* in an isolated village. The patient was a young man of nineteen, who, on the day before the attack, "walked four miles in the hot sun, with a looseness of the bowels, and bathed in a mill-pond while he was warm and perspiring. He bathed in another pond of stagnant water soon afterward. In this pool of dirty, slimy water he played about two hours, under a burning sun, and it is likely also that he swallowed some of the dirty water; he came out, and walked home in the afternoon; soon after getting home he purchased a large quantity of blue plums, unripe, and ate as many as he wanted; slept in a close, unventilated, dirty room adjoining his father's store, and was taken violently ill after midnight," 18th July, 1873. The symptoms were identical with those of a patient with Asiatic cholera, and he died in thirty hours. Dr. Alston observes: "Taking the above case upon its symptoms alone, without going into the origin or exciting cause, we would necessarily be compelled to recognize the disease to which they point. * * and with such data I am forced to the conclusion that I was dealing with cholera." He says that there was "for the past two or three summers a good deal of diarrhœa and dysentery" in the village. 1873 was a cholera year, when "people are more liable to have simple and choleraic diarrhœas, which all the authorities say if let alone are likely to terminate in cholera, especially so if they have great exciting causes as were present in the case above described." "After the death of this young man, two men were taken severely with (as I took it) cholerine or choleraic diarrhœa." They recovered, and the disease did not spread.

Taken in connection with the paper of Dr. E. M. Estrazulas, in the *American Journal of the Medical Sciences* for July, 1873, upon spontaneous epidemic cholera in Paraguay, it looks as though the accepted theory that that disease must be imported as a preliminary to every outbreak, may, like some other medical doctrines, require revision if not alteration.

